# Emerging Role of Carbon Ion Radiotherapy in Reirradiation of Recurrent Head and Neck Cancers: What Have We Achieved So Far?

**DOI:** 10.3389/fonc.2022.888446

**Published:** 2022-05-23

**Authors:** Tapesh Bhattacharyya, Masashi Koto, Paul Windisch, Hiroaki Ikawa, Yasuhito Hagiwara, Hiroshi Tsuji, Sebastian Adeberg

**Affiliations:** ^1^Department of Radiation Oncology, Tata Medical Centre, Kolkata, India; ^2^QST Hospital, National Institutes for Quantum Science and Technology, Chiba, Japan; ^3^Department of Radiation Oncology, Kantonsspital Winterthur, Winterthur, Switzerland; ^4^Department of Radiation Oncology, Yamagata University Faculty of Medicine, Yamagata, Japan; ^5^National Center for Tumor Diseases (NCT), University Hospital Heidelberg (UKHD) and German Cancer Research Center (DKFZ), Heidelberg, Germany; ^6^Department of Radiation Oncology, University Hospital Heidelberg (UKHD), Heidelberg, Germany; ^7^Heidelberg Institute for Radiation Oncology (HIRO), National Center for Radiation Research in Oncology (NCRO), UKHD and DKFZ, Heidelberg, Germany; ^8^Clinical Cooperation Unit Radiation Oncology, German Cancer Research Center (DKFZ), Heidelberg, Germany; ^9^Heidelberg Ion-Beam Therapy Center (HIT), Heidelberg, Germany

**Keywords:** carbon ion therapy, reirradiation, recurrent head and neck cancer, hadron therapy beam, radioresistance

## Abstract

Administering reirradiation for the treatment of recurrent head and neck cancers is extremely challenging. These tumors are hypoxic and radioresistant and require escalated radiation doses for adequate control. The obstacle to delivering this escalated dose of radiation to the target is its proximity to critical organs at risk (OARs) and possible development of consequent severe late toxicities. With the emergence of highly sophisticated technologies, intensity-modulated radiotherapy (IMRT) and stereotactic body radiotherapy have shown promising outcomes. Proton beam radiotherapy has been used for locally recurrent head and neck cancers because of its excellent physical dose distribution, exploring sharp Bragg peak properties with negligible entrance and exit doses. To further improve these results, carbon ion radiotherapy (CIRT) has been explored in several countries across Europe and Asia because of its favorable physical properties with minimal entrance and exit doses, sharper lateral penumbra, and much higher and variable relative biological efficacy, which cannot be currently achieved with any other form of radiation. Few studies have described the role of CIRT in recurrent head and neck cancers. In this article, we have discussed the different aspects of carbon ions in reirradiation of recurrent head and neck cancers, including European and Asian experiences, different dose schedules, dose constraints of OARs, outcomes, and toxicities, and a brief comparison with proton beam radiotherapy and IMRT.

## 1 Introduction

Using reirradiation to treat recurrent head and neck cancer is extremely challenging. These recurrent tumors are hypoxic, radioresistant, aggressive, and very close to the organs at risk (OARs). Balancing the anticipated mortality and morbidity of progressive disease and morbidity of aggressive treatment creates a dilemma in decision-making. Head and neck cancer is largely a locoregional disease, with surgical salvage being the most effective curative treatment in recurrent settings, where resection is possible for patients in good general condition. Goodwin (1) reported that the expected 5-year survival rate after salvage surgery was 39%. In previously irradiated recurrent unresectable disease, reirradiation is an alternative salvage option. Emerging evidence suggests that reirradiation can achieve reasonably fair locoregional control (LRC) in a carefully selected subset of patients; however, the achievement of expected LRC strongly depends on the reirradiation dose (2–4). Controlling these radioresistant tumors requires escalated radiotherapy doses, which are difficult to deliver because they are located close to critical OARs. Reirradiation with intensity-modulated radiotherapy (IMRT) or stereotactic body radiotherapy (SBRT) is feasible when salvage surgery is not possible (5–9). In 30%–40% of patients, reirradiation with IMRT was associated with unacceptable rates of severe toxicities (grade ≥III) (10, 11). The severity of toxicities intensified further when treatment combined photon reirradiation and chemotherapy (12, 13). However, treatment outcomes after photon reirradiation are suboptimal, with a 1-year overall survival (OS) of 30%–50% (5, 14, 15). Charged particles in the finite range are characterized by a Bragg peak with negligible entrance and exit doses. Carbon ion radiotherapy (CIRT) offers potentially superior physical dose distribution, which enables dose escalation with improved potential to spare the critical OARs. It offers greater linear energy transfer (LET) and subsequently enhanced relative biological effectiveness (RBE) that can kill more tumor cells than other radiation modalities, such as photons or protons (16). Therefore, this highly conformal and radiobiologically more effective CIRT can provide an optimized solution for treating recurrent, previously irradiated unresectable tumors with escalated radiation doses and improved toxicity profiles. This article reviews different aspects of CIRT in the reirradiation of recurrent head and neck cancers.

## 2 Current Knowledge

### 2.1 Radiobiological Aspects of Recurrent Head and Neck Cancers and Their Radioresistant Nature

The causes of locoregional failure after radical radiotherapy include the failure to sterilize all the clonogenic tumor cells, presence of hypoxic tumor cells, lack of reoxygenation, tumor cells within the cellular cycle not being optimally redistributed following radiation, and presence of inherently radioresistant tumor cells. Recurrent head and neck cancers generally originate from radioresistant clones after the initial course of curative radiotherapy or chemoradiation. Weichsalbaum et al. (17) compared the *in vitro* radiobiological parameters of tumor cell lines derived from radiotherapy failure to those of other head and neck cell lines before radiation and found that tumor cells derived from radiotherapy failures are radioresistant. Zbaren et al. (18) observed a striking presence of multicentric tumor foci in the vicinity of the gross disease. Stevens et al. (19) found that patients in whom tumors recur after primary radiotherapy can rarely be administered the identical radiation regimen again, as the tumor in the review may become radioresistant, and the healthy tissues surrounding it may not endure further doses of radiation.

Radioresistance acquired after photon radiation can have the following explanations:

1. Inducing epithelial–mesenchymal transition, i.e., phenotypical and molecular changes that cause epithelial cells to acquire characteristics of mesenchymal cells (20).
2. Enrichment of cancer stem cells (CSCs), which have greater potential to repair DNA and exhibit greater resistance to cytotoxicity induced by reactive oxygen species. Radiation treatment can enhance the relatively high presence of CSCs within tumors. Thus, enriched CSCs promote asymmetric cell proliferation (21–23).
3. Repeated photon exposure triggers overexpression of cyclin D1 and enhances cell cycle progression, causing further DNA damage and activating the Akt signaling pathway and DNA-dependent protein kinases, an element pivotal to the non-homologous end-joining (NHEJ-c) Double strand break (DSB)-repair pathway (24–26).
4. The pro-survival Akt and mammalian target of rapamycin (mTOR) signaling pathways were activated. This can ultimately boost the capacity of reirradiated cancer cells to repair DNA, thus advancing DNA acquisition (27).

### 2.2 How Do Carbon Ions Mitigate Radioresistance After Initial Radiation?

High carbon ions in LET deposit their energy along particle tracks, inducing clustered damage of higher complexity than photons. Therefore, they are biologically more efficacious. Although photons also generate a few clustered damages, their increasing frequency and complex spatial distribution induced by carbon ions make them biologically more deleterious than photons.

Several studies have highlighted the superior ability of carbon ions to kill cancer cells. This may indicate an inefficient Ku-dependent non-homologous end-joining repair pathway. Carbon ions may cause greater damage to nearby DNA structures than photons, resulting in short DNA fragments (<40 bp), preventing Ku from effectively binding to the two DNA ends, thus slowing the NHEJ-c pathway while increasing the involvement of the homologous recombination pathway.

Carbon ions also acquire the capacity for subdue resistance by escalating cell death *via* the extrinsic ceramide apoptotic pathway (28) and terminating telomerase-activated cells (29). Tumors and their microenvironment develop an adaptive response to photon radiotherapy, which may advance cellular plasticity. In non-CSCs, this induces CSC attributes and, eventually, resistance to photon radiation. High-LET irradiation can mitigate this response (30).

CIRT is highly effective for reirradiation of slowly proliferating tumors, such as adenoid cystic carcinomas (ACCs), melanomas, and head and neck soft tissue sarcomas, which have a high in review capacity for sublethal damage repair (SLDR) and a low α/β ratio and are highly resistant to conventional treatment.

CIRT can also overcome hypoxia-induced radioresistance by inducing complex DNA damage *via* densely packed ionization. It represents a unique combination of an LET in the entrance channel to a range of 11–13 kev/μm and a moderately high LET on the spread-out of the Bragg peak (SOBP) of 40–80 kev/μm (31). In comparison with photon irradiation, the sensitivity of cells to intermediate- and high-LET carbon ion irradiation depends less on oxygen tension. Thus, CIRT is thought to better control hypoxic tumors.

### 2.3 Potential Applications of Carbon Ions in Recurrent Head and Neck Cancers

#### 2.3.1 German Experience

The pilot project at GSI (Gesellschaft fürSchwerionenforschung–Helmholtzzentrum Darmstadt) adopted biologically optimized treatment plans based on local effect model I (LEM I), where the principal premise is that the local biological effect, that is, the organic damage in a small subvolume of the cell nucleus, is exclusively determined by the estimated value of the energy deposited in that sample and does not depend on the particular radiation type yielding the energy deposition. Despite its similarity to the microdosimetric approach, it applies to volumes in nano dimensions. Based on photon experience, LEMs predict the spatial distribution of particles on a nanometric scale (32). As LEM I directly links the cell nuclei’s local dose deposition pattern to the photon dose–response curve, European centers are more familiar with their practice of carbon–photon combination in both squamous and non-squamous histology. This allows for the acquisition data on reirradiation with carbon ions after photon or photon–carbon combined radiation. Europe widely practices hypofractionation, with a lesser dose/fraction compared to Japanese centers, with 5–6 days a week of treatment.

Held et al. (33) studied 229 patients with recurrent head and neck cancer who received CIRT between 2010 and 2017. Fifty-four percent of the patients had ACCs, followed by squamous cell carcinomas in around one-fourth of the patients. During reirradiation, 63% of the patients had T4 disease. Seventeen percent underwent surgical resection before CIRT, 58% received initial treatment with photon-based radiation either in the form of IMRT or three-dimensional conformal radiotherapy (3D-CRT), and 28% received initial treatment with IMRT–CIRT combination. The median total dose of CIRT was 51 Gy (RBE) [range, 36–66 Gy (RBE)] with 3 Gy (RBE) per fraction (5–6 fractions/week). The median cumulative tumor lifetime dose post-CIRT was 132.8 Gy (range, 88.8–155 Gy). None of the patients had received concurrent systemic chemotherapy. The mean OS was approximately 26 months [95% confidence interval (CI): 21.9–30.3 months]. Patients with ACC had a better median OS of approximately 33.6 months (95% CI: 22.5–44.7 months) as opposed to the median OS of 13.7 months (95% CI: 9.9–17.5 months) for individuals with squamous cell carcinoma. A smaller planning target volume (PTV) dimension at reirradiation and a radiotherapy interval of >12 months were favorable prognostic factors. The median local progression-free survival (LPFS) was approximately 24 months (95% CI: 19.4–29.0 months). The 12-month and 18-month local control (LC) rates post-CIRT, considering death as a competing risk, were 60.0% and 44.7%, respectively. Patients receiving a total dose of CIRT 51 Gy (RBE) had a notably better median LPFS of 25.5 months (95% CI: 19.9–31 months) as opposed to 16 months (95% CI: 58–26.2 months) for patients in review receiving a total CIRT dose of less than 51 Gy (RBE) (33). These encouraging data led to the prospective randomized CARE trial, which will assess toxicities and tumor control of re-IMRT vs. re-CIRT for recurrent head and neck tumors (34).

Jensen et al. in a separately performed retrospective analysis evaluated the outcomes in 52 patients who were reirradiated with CIRT between 2010 and 2013. Seventy-seven percent of the patients had T4 disease. Tumors generally developed from the paranasal sinuses (36.5%), followed by the skull base (21.2%). A total of 86.5% of the patients had macroscopic disease before reirradiation. The treated patients received a median prior radiotherapy dose of 66 Gy. Almost 27% of the patients received CIRT prior to the radiotherapy session. They received a median re-CIRT dose of 51 Gy (RBE) [range, 36–74 Gy (RBE)]. The median interval between prior radiotherapy and reirradiation was 61 months. The tumor response rate (complete and partial) was as high as 53.8%. LC at 1 year was 70.3% (2-year estimate, 47.4%), and OS at 1 year was 81.8% (2-year estimate, 63.3%). This group had a median LC of 19 months (35).

#### 2.3.2 Japanese Experience

The National Institute of Radiological Sciences (NIRS) initiated the clinical use of fast neutrons in 1974. The NIRS team was looking for depth in the carbon beam SOBP, at which neutrons would demonstrate the same RBE. LET alone does not adequately detail the energy deposition distribution around a particle track. RBE also depends on variables, such as tissue type, fractionation, and total dose. The microdosimetric kinetic model (MKM) was adopted to explain the biological effects of radiation beams based on how carbon ions stochastically deposit energy at the micrometer level (36, 37). As MKM was developed from prior neutron experience rather than a photon, no robust formula or isodose platform exists to calculate the total biologically equivalent dose by adding photon and carbon doses. NIRS and other carbon ion centers in Japan usually practice carbon-based therapy alone rather than carbon–photon combination in non-squamous relatively radioresistant histologies. Unresectable squamous cell carcinomas are traditionally treated with photon-based chemoradiotherapy with excellent outcomes. For up-front treatment of squamous cell carcinomas either in first-line or recurrent settings, Japanese clinicians do not prefer carbon ion radiation because of its radiosensitive nature and the availability of other effective treatment options. Moreover, based on their initial clinical experience, they believe that the treatment of radiosensitive squamous histology may lead to ulceration or fistula formation because of rapid and prompt regression of the tumor after CIRT. Thus, they used CIRT with or without chemotherapy to treat non-squamous histology of the head and neck. Exploiting the superior biological profile of carbon ion hypofractionation and a 4-day-a-week treatment regimen remains the hallmark of carbon ion dose regimens in Japan.

**Figure 1:**
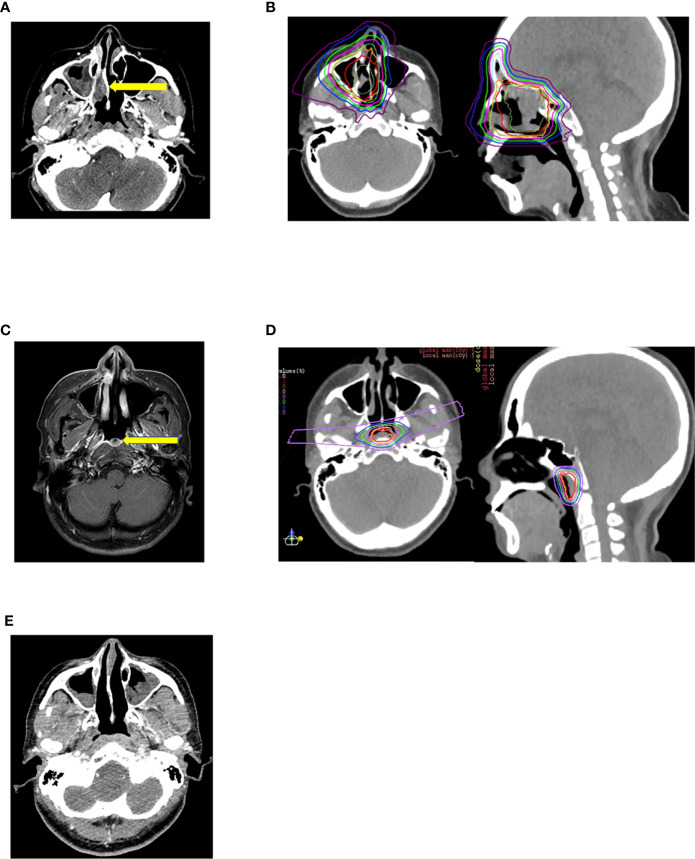
**(A)** Malignant melanoma of right nasal cavity before initial Carbon Ion Radiotherapy. **(B)** Initial carbon ion radiotherapy dose distribution. Dose delivered 64GyRBE/16fractions/4weeks. **(C)** Marginal recurrence after 7 months of initial carbon ion radiotherapy. **(D)** 2nd session of Carbon Ion Radiotherapy dose distribution. Dose delivered 57.6Gy RBE/16fractions/4weeks. **(E)** After 26 months of 2nd session of carbon ion radiotherapy patient has no evidence of disease and has not experienced any severe toxicities.

Hayashi et al. retrospectively analyzed data from 48 patients who received reirradiation treatment with CIRT at NIRS between 2007 and 2016 for recurrent head and neck malignancies. Nasal cavity or paranasal sinus tumors were present in 68.8% of the patients. All patients had a non-squamous histology, with 43.8% having malignant melanomas. Among the patients, 56.3% had stage IV disease and 22.9% had stage III disease. The most commonly prescribed dose was 52.8 Gy (RBE) in 12 fractions (43.8%) followed by 57.6 Gy (RBE) in 12 fractions (35.4%). The prescribed re-CIRT dose was 10%–20% lower than the initial CIRT dose, depending on the gap between the two radiations and proximity to critical structures. All doses were prescribed 4 times a week for 3–4 weeks, which differed from the European schedules. The median follow-up period post reirradiation for all survivors and patients was approximately 27 (range, 6.2–113.7) months and 50 (range, 11.0–133.7) months, respectively. The 2-year LC, LRC, progression-free survival (PFS), and OS rates following reirradiation were 40.5% (95% CI: 25.6%–57.3%), 33.5% (95% CI: 20.4%–49.7%), 29.4% (95% CI: 17.8%–44.4%), and 59.6% (95% CI: 45.1%–72.6%), respectively. Multivariate analysis indicated that an interval of <24 months between initial irradiation and reirradiation gave a considerably poor prediction of PFS and OS after CIRT reirradiation. They also observed that patients with marginal failure after reirradiation exhibited considerably poor prediction of PFS and LC. Representative composite **Figures 1A–E** show the outcome of a patient with recurrent malignant melanoma of the right nasal cavity treated twice with CIRT at NIRS-QST Hospital.

#### 2.3.3 Chinese Experience

Gao et al. assessed 141 patients with locally recurrent, prior irradiated head and neck malignancy who were salvaged by CIRT. Among them, 78.1% had carcinoma of the nasopharynx and 75.3% had squamous cell carcinoma of poorly differentiated or undifferentiated variety; 40% had stage IVA/IVB disease followed by stage III disease in 29.3%. A total of 91.5% of the patients were initially treated with IMRT. Before reirradiation with CIRT, 45.4% underwent chemotherapy and 16.3% underwent surgery. The median dose administered to patients who received CIRT alone was 60 Gy (RBE) [range, 50–69 Gy (RBE), 2.0–3.5 Gy (RBE)/fraction]. The median follow-up time was 14.7 months (range, 1.6–36.4 months) for the entire cohort of patients. The median time to locoregional recurrence was approximately 13 months (range, 5.9–31.1 months). With death as a competing risk, the 1-year incidence rates of local and regional control were 84.9% and 97.7%, respectively. The 1-year OS and disease-specific survival (DSS) rates were 95.9% (95% CI: 92%–99.9%) (38).

Hu et al. treated 75 patients with locoregionally recurrent, poorly differentiated, or undifferentiated nasopharyngeal cancer with carbon ion therapy at the Shanghai Proton Center between 2015 and 2017. Most patients (77.3%) had locally advanced disease at presentation during reirradiation. Most patients (96%) were initially treated with IMRT. The median initial IMRT dose was 70 Gy (range, 66–76 Gy) in 28–38 fractions. The median interval between IMRT and re-CIRT was 29 months. The median IMRT reirradiation dose was 57.5 Gy (RBE) [range, 50–66 Gy (RBE)] at a dose of 2–3 Gy (RBE) per fraction. Induction chemotherapy was administered to 61.3% of the patients, whereas concurrent chemotherapy was delivered to only 16% of the patients. Over a median follow-up time of 15.4 months, the 1-year OS and DSS rates were 98.1% and the 1-year local recurrence-free survival rate was 86.6% (39).

#### 2.3.4 Italian Experience

Between 2013 and 2016, 51 successive patients with inoperable recurrent salivary gland tumors were treated with CIRT at Centro Nazionale Adroterapia Oncologica (CNAO), Italy. Most patients (74.5%) had ACCs, and 51% had rcT4a disease, followed by rcT4b disease in 37% of the cases. The median prior photon-based dose was 60 Gy. The median re-CIRT dose was 60 Gy (RBE) with 3.0–5.0 Gy (RBE) per fraction, 4–5 fractions per week, with 72 Gy (range, 37.5–138.1) as the median biological effective dose (BED). All patients had gross disease during CIRT in 42 cases (82.4%) observed in the high-dose region of the previous photon-based RT. The median duration of the gap between the two sessions of radiation was 6.33 years. At a median follow-up period of 19 months (range, 2–57 months), LC was 41.2% at the last follow-up. The estimated PFS (actuarial) at 1 and 2 years was 71.7% and 52.2%, respectively. The estimated OS (actuarial) at 1 and 2 years was 90.2% and 64.0%, respectively. Gross tumor volume (GTV), female sex, and interval between two radiation sessions were significant prognostic factors for OS (40). A global comparison of landmark carbon ion reirradiation experiences is displayed in **Table 1**.

**Table 1:** Comparison of different re-CIRT approaches from established CIRT centers.

Studies	Japan NIRS	Heidelberg Germany	Heidelberg Germany	CNAO Italy	Shanghai China
	Hayashi et al. (41)	Held et al. (33)	Jensen et al. (35)	Vischioni et al. (40)	Gao et al. (38)
Type	Retrospective	Retrospective	Retrospective	Retrospective	Observational study
Period	2007–2016	2010–2017	2010–2013	2013–2016	2015–2017
Sample size	48	229	52	51	141
Location predominantly at reirradiation	Paranasal sinuses: 37.5% Nasal cavity: 18.8%	Salivary gland: 24% Nasopharynx: 22.7% Paranasal sinus: 21.4%	Paranasal sinus: 36.5% Base of skull/intracranial: 21.2% Parotid gland: 19.2%	Parotid gland: 33.3%	Nasopharynx: 78.1% Nasal cavity/Paranasal sinus: 8.2%
Staging at reirradiation	Stage III: 22.9%	T3: 8.3%	T3: 19.2%	T4a: 51%	Stage III: 29.3%
	Stage IV: 56.3%	T4: 62.5%	T4: 76.9%	T4b: 37%	Stage IVA/IVB: 40%
Initial radiation	CIRT: 100%	IMRT: 35% 3D-CRT: 23% IMRT and CIRT boost: 28%	CIRT: 27% Photon based: 73%	Photon based: 100%	IMRT: 91.5% SRS: 0.7%
Simultaneous chemo or immunotherapy	Concurrent-No	Concurrent-No	Concurrent-No	Concurrent-No	Concurrent-No Induction-Yes: 45.4%
Salvage surgery before re CIRT	No	Yes: 17% No: 83%	Yes: 13.5% No: 86.5%	No: All were inoperable cases	Yes: 16.3%
Histology	Malignant melanoma 43.8% Adenoid cystic carcinoma 35.4%	Adenoid cystic carcinoma: 54.1% Squamous cell carcinoma: 26.2%	Adenoid cystic carcinomas: 74.5% Mucoepidermoid carcinomas: 11.8%	Adenoid cystic carcinoma: 74.5% Mucoepidermoid carcinoma: 11.8%	Squamous cell carcinoma: 75.3% Adenoid cystic carcinoma: 6.8%
Initial RT dose	48 Gy RBE–70.4 Gy RBE In 12-16#; most common 57.6 Gy RBE/16#	Median IMRT dose 50 Gy/25# (Range 45–56 Gy); Median CIRT boost 24Gy RBE (Range 18–24 Gy RBE)	Median prior radiotherapy dose 66 Gy	Not reported	Not reported
Prescribed dose for re-CIRT	Median 54 Gy (RBE) [Range 40–64 Gy (RBE)]	Median 51 Gy (RBE) [Range 36–66 Gy (RBE)]	Median 51 Gy (RBE) [Range 36–74 Gy (RBE)]	Median 60 Gy (RBE) [Range 45–68 Gy (RBE)]	Median 60 Gy (RBE) [range 50–69 Gy (RBE)]
Dose per fraction	4 Gy (RBE)/fraction	3 Gy (RBE)/fraction	3 Gy (RBE)/fraction	3–5 Gy (RBE)/fraction	2–3.5 Gy (RBE)/fraction
Target volume delineation	CTV = GTV+5 mm PTV = CTV+2 mm Elective nodal areas not included.	CTV = GTV+2–5 mm PTV = CTV+2–3 mm Elective nodal areas are not included.	Target volume = GTV+2 mm safety margin. Elective nodal areas not included	CTV = GTV+0–5 mm, microscopic extension of tumor along the nerves PTV = GTV+2 mm	CTV = GTV+3–5 mm PTV = CTV+1–3 mm
Biophysical Model used	Microdosimetric kinetic model	Local effect model version 1	Local effect model version1	Local effect model version 1	Local effect Model 1
Beams used	2 pairs of orthogonal beams (Vertical and horizontal beams)	Usually horizontal beams	Horizontal or vertical beams depending on tumor location	NA	2–3 treatment portals from primary horizontal beams
Method	Passive beam or spot scanning	Raster scanning	Raster scanning	Spot scanning	Raster scanning
Gap between 2 radiation treatment	Median 24.2 months (range, 4.5–112.5) months	Median 3.9 years (range, 0.3–46.5 years)	Median 61 months (range, 9–620 months)	Median 6.33 years (range, 1.08–20 years)	Median 36 months (range, 11–257 months)
Dose constraints	Spinal cord: Dmax 30 Gy (RBE) Brain stem: Dmax 30 Gy (RBE) Optic nerve: D20 <40 Gy (RBE) Optic chiasm Dmax 30 Gy (RBE)	QUANTEC Guidelines (Mentioned in **Table 2**)	Cumulative dose to the brainstem and spinal cord was kept below 60 Gy and 50 Gy, respectively, assuming around 50% recovery of the CNS	QUANTEC and NIRS dose constraints	Emami et al. (42) NIRS dose constraints
Target volume Median (Range)	GTV = 10.4 cc (0.5–89.5 cc)	CTV = 85.2 cc (6.3–710.5 cc) PTV = 128.9 cc (13.3–925 cc)	CTV = 93 cc (6–618 cc)	GTV = 28.58 cc (1.75–205.54 cc) CTV = 76.56 cc (2.74–865 cc)	Not mentioned
Operability at reirradiation	Inoperable: 81.3% Operable: 18.7%	Salvage surgery before CIRT: 17%	Prior salvage surgery: 13.5%	Not mentioned	Not mentioned
Follow-up	Median 27.1 months	Median 28 months	Median 14 months	Median 19 months	Median14.7 months
LRC	2-year local control 40.5% 2-year locoregional control 33.5%	1-year local control 60% 18 months local control 44.7%	1-year local control70.3% 2-year estimate 47.4%	Estimated PFS 1-year 71.7% 2-year 52.2%	1-year local control 84.9% 1-year regional control 97.7%
OS	2-year 59.6%	1-year 72% 18 months-59.2%	1-year 81.8% 2-year estimate 63.3%	Estimated OS 1-year 90.2% 2-year 64%	1-year 95.9%
Acute toxicities	Overall grade III-10.4% Grade III mucositis 8.3% Grade III dermatitis-2%	Grade ≥III -3.1%	Grade II mucositis 11.5% Grade II dermatitis 9.6%	Grade III-3.9%	Grade IV acute hemorrhage 2 cases
Late severe toxicities	Overall Grade ≥III 37.5%	Overall Grade ≥III 14.5%	Overall Grade ≥III 17.1%	Overall Grade III 17.5%	Overall Grade III 10.6%
CNS necrosis	Grade IV-2% Grade V-2%	Grade III-4% Grade IV-0.8%	Grade III-3.8%	No grade III CNS necrosis	Grade III 0.7%
Optic nerve disorder	GradeIV-18.75%	Grade ≥III -1.7%	NA	Grade III visual defect 5.8% Grade III neuropathy 1.9%	Grade III cranial nerve disorder 2.1%
Soft tissue necrosis	Grade III-0%	Grade III -0%	Grade III-3.8%	Grade III-0%	Grade III 7.1%

IMRT, intensity-modulated radiotherapy; 3D-CRT, three-dimensional conformal radiotherapy; SRS, stereotactic radiosurgery; CIRT, carbon ion radiotherapy; RBE, relative biological effectiveness; GTV, gross tumor volume; CTV, clinical target volume; PTV, planning target volume; QUANTEC, quantitative analysis of normal tissue effects in the clinic; NIRS, National Institute of Radiological Sciences; LRC, locoregional control; OS, overall survival; CNS, central nervous system; PFS, progression-free survival. NA, Not available.

### 2.4 Dose Constraints at Carbon Ion Radiotherapy Reirradiation

The dose constraints for OARs during reirradiation with carbon ions are variable and complex. Jensen et al. (35) in their report of reirradiation with CIRT for ACC maintained the cumulative dose to the brain stem below 60 Gy and to the spinal cord below 50 Gy, anticipating 50%−70% recovery of the neural structures depending on the time interval between two sessions of radiation, as described by Ang et al. (43). They may have considered more than 50% recovery of the CNS, as the median gap between the two sessions of radiation was 3.9 years. In this report, the mode of radiation that 58% of the patients initially received was photon-based in the form of either IMRT or 3D-CRT (37). These experiences led to conservative constraints assuming recovery of at least 20%–30% in neuronal structures after initial radiotherapy, stratified by time interval. The projected radiation tolerance of OARs for re-CIRT, in accordance with the Quantitative Analyses of Normal Tissue Effects in the Clinic (QUANTEC) guidelines and German experience (36, 44, 45), is displayed in **Table 2**. While assessing recuperation from occult spinal cord injury in their study, Ang et al. (43) established that following initial doses of 45 Gy to the spinal cord, reirradiation of 65–68 Gy in conventional fractionation is well within tolerance. Furthermore, for toxicities to the brain stem and spinal cord, the dose applied and the volume exposed to high-dose radiation therapy are both relevant. For carbon ions, the spinal cord shows distinctly different tolerance to radiation. Observation of the endpoint ‘‘symptomatic myelopathy” in rats treated with a single fraction and fractionated photons along with Bragg peak and plateau carbon ions demonstrates that the repair processes after fractionated photon treatments may be identical for plateau carbon ions but were notably reduced in Bragg peak carbon ions (46). For fractionated carbon ion treatments with six and 18 fractions, Bragg peak carbon ions have an increased association with side effects induced by radiation in the rat spinal cord and then to the plateau region (47). Despite these findings, the extent of extrapolating experimental data to clinical settings is obscure in most clinical situations. Hayashi et al. (41) used the same dose constraints during re-CIRT for the brain stem and spinal cord [30 Gy (RBE)] and for the optic nerve 40 Gy (RBE), wherever possible, as they used during up-front radiation with carbon ions. This was feasible, which may be due to the excellent conformal dose distribution and extremely sharp lateral penumbra of carbon ions with negligible entrance and exit doses. They reported a median interval of approximately 2 years between the initial radiotherapy and reirradiation. Unlike photons, data regarding the tolerance and dose constraints of the spinal cord with re-CIRT are lacking. Based on the radiobiological conclusions by Nieder et al. (48), Hu et al. (38) set the recovery percentage from the prior IMRT dose at 70%. They followed the dose constraints described by Emami et al. (42) and also adopted dose constraints based on previous experience with NIRS in Japan (49). Their brain stem dose constraint was relatively higher at 45 Gy (RBE) than at NIRS constraints. Gao et al. (38) from Shanghai used a similar dose constraint to that of NIRS, although treatment planning followed the optimization of biological treatment plans exploring LEM. Vischioni et al. (40) derived from the CNAO set constraints on OARs using QUANTEC guidelines, except for the brain stem, spinal cord, and optic nerve, which were derived from previous experiences from NIRS (49). Interestingly, most of the centers followed the NIRS dose constraints for the optic nerve, spinal cord, and brain stem, although the dose per fraction and biophysical models used were different (LEM I in Europe vs. mixed beam or MKM in Japan). In the NIRS approaches (both MKM and mixed beam), the RBE was first obtained from their neutron beam experience, where the biological endpoint was set at 10% survival of human salivary gland cells. In LEM, the calculation of RBEs applies to any fraction size rather than to a particular survival fraction. Predictions by the two models differed with the same prescribed biological dose. Any LEM follower intending to apply an NIRS dose schedule should introduce corrections (50–52). Moreover, the initial radiation modality used in most European centers was photon-based or a photon–carbon combination, whereas in Japan, CIRT alone was used in both sessions, and the median duration between the two sessions of radiation also varied widely among institutes.

**Table 2:** Dose constraints of OARs as proposed in the CARE trial by Held et al. (34).

Structures	Maximum Cumulative EQD2 (RT interval ≤24 months)	Maximum Cumulative EQD2 (RT interval >24 months)	Comments
Brain stem *(α/β = 2)*	60	72 (≙+20%)	Maximum (surface)
Optic chiasm *(α/β = 3)*	54	64.8 (≙+20%)	Maximum
Optic nerves *(α/β = 3)*	54	64.8 (≙+20%)	Maximum
Spinal cord *(α/β = 2)*	50	60 (≙+20%)	Maximum
Further OARs	ALARA		/

OAR, organ at risk; EQD2, equivalent dose in 2-Gy fractions; RT, radiotherapy; ALARA, as low as reasonably achievable.

### 2.5 Target Volume Delineation Pattern

Regardless of the histology and the subsites of the head and neck, a similar contouring pattern during re-CIRT was observed across different countries and institutes. Jensen et al. in their re-CIRT target volume incorporated only the visible local relapse with a small safety margin of 2 mm and did not perform any elective nodal irradiation. No separate clinical target volume (CTV) was reported in their study (37). In the study by Held et al. from Heidelberg, the CTV comprised a visible tumor on contrast-enhanced CT or MRI with a 2−5-mm safety margin. CTV included the resection cavity in patients who underwent prior (partial) surgical resection. The CTV comprised only involved lymph nodes, and no elective lymph nodal irradiation was performed (35). Hayashi et al. defined CTV as GTV plus a 0–5-mm margin. The CTV was trimmed from the OARs in cases where it was near the OAR. PTV was defined as CTV plus a 2-mm margin for safety against setup mistakes and uncertainties (41). In a study by Gao et al. from Shanghai, the CTVs comprised the GTV plus a margin of 3–5 mm to record potential microscopic spread. Smaller CTV margins were permitted for lesions near critical OARs that were irradiated earlier. Prophylactic irradiation for subclinical disease to any uninvolved region was not administered regardless of the probability of disease involvement. The CTV was given an extra 1–3-mm margin to create the PTV (38). Hu et al. from Shanghai used a more liberal margin for the reirradiation of nasopharyngeal carcinomas. The CTVs of both the GTV of the primary site and neck were designed to include 5 mm beyond the GTV for microscopic extension (limited to 1 mm near the OAR) and a variable margin for occult tumor spread. An extra 3–6-mm margin was included in the CTV to create the PTV. For patients with tumor shrinkage after chemotherapy, prechemotherapy tumor volumes were assigned a universal definition of subclinical disease for inclusion in the CTV (39).

### 2.6 Optimal Dose Fractionation Scheme for Carbon Ion Reirradiation

Various dose fractionation schedules have been used in studies. Several investigators have gradually escalated the dose and attempted to determine the optimum dose for reirradiation. The median reirradiation dose in all studies varied between 51 and 60 Gy (RBE). Both German studies used a median reirradiation dosage of 51 Gy (RBE) at 3 Gy (RBE), 5–6 days a week (35, 37). Jensen et al. achieved 132.8 Gy as a median cumulative dose (37). Hayashi et al. reduced the 10% dose from initial reirradiation, and the most commonly followed regimens were 52.8 Gy (RBE)/12 fractions (43.8%), followed by 57.6 Gy (RBE)/12 fractions (35.4%). The dose per fraction was much higher 4.6–4.8 Gy (RBE), and all patients were treated with four fractions in a week. The linear-quadratic model enables them to achieve a median cumulative equivalent dose in 2-Gy fractions (EQD2) of 136.3 Gy (RBE) (41). Italian studies used a mixed Japanese–German approach for salivary gland tumors, where they delivered a relatively higher median dose of 60 Gy (RBE) at 3–5 Gy (RBE)/fraction in 4 weekly fractions with a median BED value of 72 Gy (RBE) (40). Chinese clinicians mostly treat nasopharyngeal cancers and may escalate the dose from 50 Gy (RBE) to reach a median dose of 60 Gy (RBE) at 2–3.5 Gy/fraction, 5 days a week (38, 39). From these studies, the optimum dose fractionation schedule that should be followed is unclear. To maximize the expected tumor control and sparing OARs, each patient’s fractionation schedule was selected based on pathology, tumor location and size, and prior radiation dosage. It also depends on the method of prior radiation used, the interval between two sessions of radiation, the biological model used, and the volumes of the target in the initial and reirradiation sessions.

### 2.7 Patterns of Failure After Carbon Ion Reirradiation

Most disease progression after reirradiation was local relapse, either solitary or combined with distant metastasis. Of these local failures, the vast majority were infield recurrences, with few marginal and in the dose gradient region close to the optic apparatus or other OARs. This suggests that there is still room for improvement in the control of local failures. However, further dose escalation is a challenging task, which may lead to higher soft tissue necrosis and vascular injuries. Mapping of dose-averaged LET within the target volume and its correlation with infield recurrence will help us guide LET optimization within the target and create a biological dose and dose-averaged LET-optimized reirradiation plan to achieve optimum results (58).

### 2.8 Acute Toxicities

The most commonly reported acute toxicities were grade I/II mucositis and dermatitis. The vast majority of patients in all studies completed the scheduled treatment without interruption. As only gross tumors with very small safety margins were treated without considering the elective nodal regions, acute reactions were more localized and concentrated, and grade III reactions appeared near the end of the treatment. Hayashi et al. (41) reported overall grade III acute toxicities in 10.4% of the patients, whereas Jensen et al. (35) reported no grade III acute toxicities in their patients. The remaining studies reported grade III acute reactions in less than 5% of the patients. Most of the acute toxicities in all studies resolved within 6–8 weeks of CIRT completion (35, 37–41). Two patients assessed by Held et al. (35) developed grade IV laryngeal edema, resulting in emergency tracheostomy and endotracheal intubation, respectively. The rate of acute reactions in all of the studies is displayed in **Table 1**.

### 2.9 Late Toxicities

Late toxicities after reirradiation depend on the number of target volumes, tumor location, interval between the two radiations, and length of follow-up duration. The most common late toxicities noted in most CIRT studies were brain necrosis, visual defects, cranial neuropathy, and soft tissue necrosis. The most serious complications after carbon ion reirradiation were soft tissue necrosis and carotid artery hemorrhage. Most of these CIRT studies reported severe grade ≥III late toxicities in 14%–20% of the patients (33–35, 38, 39, 41), whereas Hayashi et al. (41) reported the highest overall grade ≥III toxicities of 37.5% potentially because of a longer follow-up (27.1 months), a relatively shorter interval between two radiation sessions (24.2 months), and a higher dose per fraction. Jensen et al. reported that overall late toxicity was modest despite the median follow-up of 14 months being comparatively short. Two patients (3.8%) experienced only grade IV internal carotid artery hemorrhage after tissue necrosis in the nasopharynx following aggregated doses of 149 and 182 Gy BED (35).

Vision loss was mostly observed in patients in whom the tumor involved or abutted the optic apparatus. Target coverage in these patients was given maximum priority as compared to maximum constraints of the ipsilateral optic nerve after discussion and obtaining consent of the patients. All studies, except that of Vischioni et al. (40), reported carotid artery hemorrhage in a small proportion of patients (<5%). Vischioni et al. avoided this complication completely because tumors were mainly located in the parotid region compared to the nasal cavity, paranasal sinus, or nasopharynx in other studies and followed the carotid-sparing technique with separate carotid artery constraints. Interestingly, Held et al. found an incidence where the temporal lobe had 16.6% blood–brain barrier disruption after re-CIRT in head and neck tumors. Mean Maximum dose (Dmax) re-CIRT to the affected cerebral area was 53.2 Gy (RBE) [range, 3.3−64.0 Gy (RBE)]. The median time to the first occurrence of radiation necrosis grades I, II, and III was 9.2, 10.2, and 16.6 months, respectively (35). Therefore, dose constraints to unaffected brain areas, particularly the temporal lobes, should be considered (59). The patterns of late toxicity are shown in **Table 1**.

### 2.10 Comparison With Proton Beam Therapy and Intensity-Modulated Radiotherapy

With advanced radiotherapy, techniques such as IMRT and SBRT, reirradiation has emerged as a potential curative treatment. Although IMRT and SBRT provide excellent conformal dose distribution, relatively lower isodoses are spread over a wide range, as photons have an infinite range and higher entrance and exit doses. Conversely, the unique Bragg peak attributes of proton beam radiation therapy (PBRT) deliver a higher dose to the tumor but minimize delivery to the normal tissues that were irradiated earlier. PBRT has the dual capacity of maximizing the focused radiation dose to the tumor and minimizing its exit dose to adjacent OAR. In addition to its excellent physical dose distribution and Bragg peak property, carbon ion is gifted with a biological advantage because of its much higher and variable RB. SBRT-IMRT is preferred in squamous histology, whereas particle therapy has shown excellent outcomes in non-squamous histology, such as ACCs, adenocarcinomas, or malignant melanomas. There are no head-on comparisons between these three modalities for recurrent head and neck cancer reirradiation. Results of proton beam therapy and IMRT in re-irradiation of head and neck cancers are displayed in **Table 3**.

**Table 3:** Results of proton and IMRT comparison in reirradiation of head and neck cancer.

Studies	Modality	Sample size	Histologies	Locoregional control	Overall survival	Toxicities
Karam et al. (53)	SBRT	18	Squamous 39% Non-squamous 61%	2-year LRC 53%	2-year OS 39%	Acute Grade IV–V 3% Late Severe soft tissue necrosis 22%
Takiar et al. (10)	IMRT	206	Squamous 84% Non-squamous 16%	2-year LRC 59%	2-year OS 51%	Late Grade III 32% at 2 years 48% at 5 years
Romesser et al. (54)	Proton	92	Squamous 56.5% Non-squamous 44.5%	1-year LC 25.1%	1-year OS 65.2%	Acute Grade III mucositis 9.9% Grade III dysphagia 9.1% Grade III dermatitis 3.3% Late Grade III dermatitis 8.6% Grade III dysphagia 7.1% Grade V bleeding 2.9%
Phan et al. (55)	Proton	60	Squamous 66.7% Non-squamous 33.3%	1-year LFFS 68.4% 2-year LFFS 55.9%	1-year OS 81.3% 2-year OS 69%	Acute Grade III dermatitis 13.3% Grade III mucositis 10% Grade III dysphagia 5% Late Grade III toxicities 20%
McDonald et al. (56)	Proton	61	Squamous 52.45% Non-squamous 48.55%	2-year local failure 19.7%	2-year OS 32.7%	Acute Grade III dermatitis 4.9% Grade III mucositis 3.3% Late Grade III or more 22.9%
Yamazaki et al. (57)	Charged Particle/SBRT and IMRT	CP 26 SBRT 117 IMRT 33	CP Squam /Non-squamous 46%/54% Photon Squam/Non-squamous 93%/7%	CP 1-year LC 66.9% Photon 1-year LC 67.1%	CP 1-year OS 67.9% Photon 1-year OS 54.1%	Overall toxicities Grade III Photon 14% vs. CP 19% Grade IV Photon 1% vs. CP 12% Grade V Photon 9% vs. CP 15%

SBRT, stereotactic body radiotherapy; IMRT, intensity-modulated radiotherapy; LFFS, local failure-free survival; CP, charged particle; LC, local control; OS, overall survival; LRC, locoregional control.

Takiar et al. (10) performed a retrospective analysis of 227 patients who were reirradiated using IMRT between 1999 and 2014. Treatment with curative intent was administered to 91% of the patients. Most patients (76.2%) had a squamous histology. Fifty percent of the patients underwent salvage resection. For squamous cell carcinomas, the 2-year OS was 51% and the LRC rate was 59%. Grade 3 toxicity at 2 and 5 years had actuarial rates of 32% and 48%, respectively, with odynophagia or dysphagia being the most common.

Karam et al. (53) reported on 18 patients who received SBRT for recurrent salivary gland tumors. Most patients did not undergo surgical resection, and all patients had positive margins. The median SBRT dose was 30 Gy administered in five fractions, with a median cumulative dose of 91.1 Gy. The 2-year PFS, LC, and OS rates were 24%, 53%, and 39%, respectively.

Vargo et al. (60) performed a retrospective analysis of the outcomes of 132 patients who received SBRT ± cetuximab treatment for locally recurrent previously irradiated head and neck cancer. The 1-year actuarial OS and LRC rates were 38% and 48%, respectively. Overall, toxicity rates among patients remained low, 12% and 7% experienced severe (grade III), acute, and late toxicity, of which the majority were related to skin and mucosal reactions. Discussing all IMRT and SBRT studies in the reirradiation of head and neck cancer is beyond the scope of this study.

Although recurrent head and neck cancer is one of the commonly cited indications for PBRT, there are few studies regarding the role of PBRT in reirradiation of recurrent head and neck cancer.

In a multi-institutional study, Romesser et al. assessed the outcomes of 92 patients who received PBRT; initial radiotherapy and PBRT had a median gap of 34.4 months. The cohort was heterogeneous in terms of tumor location. The cumulative incidence of locoregional failure at 12 months, considering death as a competing risk, was 25.1% with a 12-month OS of 65.2%. Grade III or greater late skin toxicities and dysphagia occurred in 8.7% and 7.1% of the patients, respectively. Two patients experienced grade 5 toxicities secondary to iatrogenic hemorrhage (54).

Phan et al. reirradiated 60 patients with recurrent head and neck cancer with squamous and non-squamous histology. Radiation treatments had a median interval of 47.1 months. Fifty-eight percent of the patients underwent up-front surgery, while 73% underwent concurrent chemotherapy. The 1- and 2-year local failure-free survival rates were 68.4% and 55.9%, respectively. Thirty percent of the patients experience acute grade III toxicities (55).

McDonald et al. reported the results of 61 patients treated with PBRT, mainly for tumors of the skull base structures, at a median gap of 23 months after the most recent prior radiation course. Of the patients, 47.5% underwent salvage surgery before reirradiation. Gross macroscopic disease was found in 70.5% of the patients. The 2-year overall rate was 32.7%, and the median OS was 16.5 months. The 2-year cumulative incidence of local failure considering death as a competing risk was 19.7%, with a regional nodal failure rate of 3.3%. Grade ≥III toxicities were acute and late in 14.7% and 24.6% of the patients, respectively (56).

Recent reports by Yamazaki et al. suggest improved results with particle therapy compared to photon therapy when reirradiating recurrent cancers. The LC rates for patients treated with photon radiotherapy and charged particles at 1 year were 67.1% and 66.9%, respectively. The 1-year OS rates were 54.1% for photon radiotherapy (55% for CyberKnife and 51% for IMRT) and 67.9% for charged particles. Twenty-seven percent of the patients presented with grade 3 or higher toxicity (24% administered photon radiotherapy: IMRT, 23%; Cyberknife, 21%; 46% of the patients were treated with charged particles; p = 0.04) (57).

In a multicenter *in silico* trial, Eekers et al. (61) compared photon, proton, and carbon ion plans for reirradiating patients with Head and Neck Squamous cell carcinoma (HNSCC). The ROCOCO trial achieved a diminution in the mean dose to OARs using particle therapy as opposed to photons in the reirradiation of HNSCC. Favoring carbon ions above PBRT provides a dosimetric advantage. In most cases, intensity-modulated ion therapy yielded a greater advantage than intensity-modulated proton therapy possibly because of different beam attributes, such as smaller spot size or sharper lateral penumbra. Such reduced doses may potentially have lower rates of severe complications related to reirradiation. Another clinical scenario in which PBRT may be a valuable approach is therapy de-escalation, for example, in human papilloma virus (HPV)+ HNSCC and unilateral neck irradiation. Here, unilateral PBRT can offer adequate coverage of the PTV while sparing the contralateral neck, which maintains the in review possibility of applying a second radiotherapy session to the contralateral neck in cases of recurrence or a second head and neck malignancy.

## 3 Future Directions

The biological effect of CIRT depends not only on the physical dose but also on the dose-averaged LET distribution. Inaniwa et al. (62) have proposed intensity-modulated composite particle therapy, which offers optimization of dose and LET distributions within the target volume. This LET painting attempts to confine the high-LET component to the GTV, which is already irradiated, hypoxic, and rich in radioresistant clones, while employing a reduced LET segment to normoxic tissues. An optimally distributed dose and dose-averaged LET within the irradiated target volume using multiple ions, such as carbon, protons, helium, and oxygen, will provide the ultimate solution for controlling such radioresistant tumors with acceptable toxicities. Ultrahigh-dose rate No expandable full form. It is ultra high dose rate radiotherapy (FLASH) CIRT can achieve a better therapeutic ratio by creating a highly oxygenated environment around the irradiated hypoxic tumor while simultaneously protecting the critical OARs (63). In addition to technological improvements, prospective trials are required to show the added benefit of carbon ions vs. protons or SBRT in terms of improved tumor control and reduced toxicities. Such a trial will aid in identifying the optimal target population for CIRT in treating such challenging tumors.

## Author Contributions

All authors have equally contributed in designing the review, data collection, and article writing. All authors contributed to the article and approved the submitted version.

## Conflict of Interest

SA received grants from Accuray International Sàrl and Merck Serono GmbH, Novocure GmbH, MSD, and Astra Zeneca outside the submitted work. PW received grants from Bridge-Proof of concept 195054 (Swiss National Science Foundation) that is outside the submitted work. He also planned for patents on methods for the detection of neurological abnormalities that are not related to the submitted work.

The remaining authors declare that the research was conducted in the absence of any commercial or financial relationships that could be construed as a potential conflict of interest.

## Publisher’s Note

All claims expressed in this article are solely those of the authors and do not necessarily represent those of their affiliated organizations, or those of the publisher, the editors and the reviewers. Any product that may be evaluated in this article, or claim that may be made by its manufacturer, is not guaranteed or endorsed by the publisher.
